# Supplementary material for: The relationship between transmission time and clustering methods in Mycobacterium tuberculosis epidemiology
Source: eBioMedicine. 2018 Oct 16;37:410–6. doi: 10.1016/j.ebiom.2018.10.013 (PMC6284411; doi:10.1016/j.ebiom.2018.10.013)
Supplement: Supplementary file 1 — Supplementary material [file mmc1.pdf]

Supplementary material

Convergence removal

For the classical genotyping methods (Spoligotyping and MIRU-VNTR), convergence of patterns can occur [1,2] resulting in incorrect clustering. Such patterns were removed based on inter-sub-lineage convergence across the phylogenetic tree. Firstly, the SNP alignment of all isolates was used as the basis for creating a maximum likelihood (ML) phylogeny. RAxML-NG version 0.5.1b[3] was used to reconstruct the phylogeny from this alignment using a GTR+GAMMA model of evolution, accounting for ascertainment bias[4] with the Stamatakis reconstituted DNA approach[5] and site repeat optimisation[6] with 20 different starting trees and 100 bootstraps. All subsequent topology visualisation was undertaken using GraPhlAn[7]. Mtbc lineage and sub-lineage numbering was then applied to all isolates based on the Coll SNP set[8]. If the same clustering pattern was observed in two different sub-lineages, with other patterns seen in-between on the tree, this was flagged as pattern convergence. For example, if isolates with the same Spoligotyping pattern appeared in lineage 4,1 and 4,6 with different patterns in-between, this was confirmed as a convergent pattern.

Convergent evolution was found to affect 39% (12) of Spoligotyping-based clusters and 16% (6) of the MIRU-VNTR clusters. Convergence-free versions of these methods (Spoligotyping, MIRU-VNTR and the combination of both) were then used as input to BEAST2 for divergence dating, as outlined in the main methods. Supplemental table 1 outlines their median transmission ages alongside the 95% HPD.

Supplemental table 1: Clustering method overview with convergent patterns removed from classical methods.

For each clustering method, the general features are outlined in the table. Median ages and 95% HPD ranges are based upon the BEAST-2 estimates of clade heights (see methods).

Method	Strains in clusters	Number of clusters	Percent of strains in clusters	Cluster sizes	Maximum SNP distances	Clustering rate	Mean Timespan	Timespan 95% HPD
Spoligotyping	118	21	36.42	2-28	0-189	0.2994	76.51	0.81 - 823.21
MIRU-VNTR	121	32	37.35	2-11	0-48	0.2747	26.08	0 - 162.27
Spoligotyping-MIRU-VNTR	50	12	15.43	2-10	2-48	0.1173	32.92	0.8 - 216.31
1 SNP cluster	74	29	22.84	2-6	0-2	0.1389	3.91	0 - 23.54
5 SNP cluster	147	40	45.37	2-27	0-10	0.3302	10.86	0 - 47.07
12 SNP cluster	242	47	74.69	2-34	0-23	0.6019	23.63	0 - 102.58
1 allele cgMLST	80	31	24.69	2-6	0-4	0.1512	4.73	0 - 24.65
5 allele cgMLST	173	42	53.4	2-28	0-22	0.4043	13.4	0 - 68.53
12 allele cgMLST	254	45	78.4	2-39	0-51	0.6451	24.06	0 - 112.25

1) 2) 3)

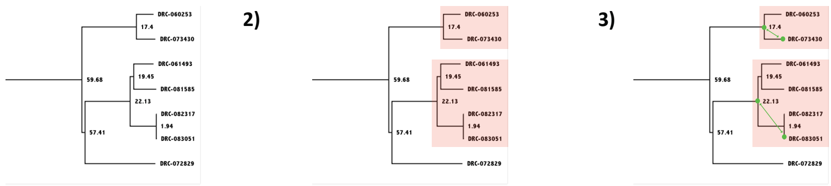

4)

Cluster	Age 1
1	13
2	20

5)

Cluster	Age 1	Age2	...
1	13	11	...
2	20	19	...

6)

Clustering approach
Median: 12
95% HPD: 10-20

Supplemental figure 1. Algorithm for estimating transmission times encompassed by different clustering approaches.
Step 1: Extract the tree from the MCMC sampled step.
Step 2: Map the clusters on the tree.
Step 3: Get the time difference between the ancestral node (most recent common ancestor) and the youngest (furthest from the ancestor) sampled tip in each cluster. These are defined as the age of each cluster.
Step 4: Aggregate all these ages across clusters.
Step 5: Repeat for every tree calculated in each MCMC sampled step.
Step 6: Calculate the median and 95% HDP based on all the ages of all clusters in all MCMC steps for the given clustering approach.

Supplemental references

1. Scott AN, Menzies D, Tannenbaum T-N, Thibert L, Kozak R, Joseph L, et al. Sensitivities and specificities of spoligotyping and mycobacterial interspersed repetitive unit-variable-number tandem repeat typing methods for studying molecular epidemiology of tuberculosis. J Clin Microbiol. 2005 Jan;43(1):89–94.
2. Driscoll JR. Spoligotyping for Molecular Epidemiology of the Mycobacterium tuberculosis Complex. In: Methods in molecular biology (Clifton, NJ). 2009. p. 117–28.
3. Kozlov A. RAxML-NG. 2017.
4. Lewis PO. A likelihood approach to estimating phylogeny from discrete morphological character data. Syst Biol. 2001;50(6):913–25.
5. Leaché AD, Banbury BL, Felsenstein J, de Oca A nieto-M, Stamatakis A, K. S, et al. Short Tree, Long Tree, Right Tree, Wrong Tree: New Acquisition Bias Corrections for Inferring SNP Phylogenies. Syst Biol. 2015 Nov;64(6):1032–47.
6. Kobert K, Stamatakis A, Flouri T. Efficient Detection of Repeating Sites to Accelerate Phylogenetic Likelihood Calculations. Syst Biol. 2016 Aug 29;66(2):syw075.
7. Asnicar F, Weingart G, Tickle TL, Huttenhower C, Segata N. Compact graphical representation of phylogenetic data and metadata with GraPhlAn. PeerJ. 2015 Jun 18;3:e1029.
8. Coll F, McNerney R, Guerra-Assunção JA, Glynn JR, Perdigão J, Viveiros M, et al. A robust SNP barcode for typing Mycobacterium tuberculosis complex strains. Nat Commun. 2014 Jan 1;5:4812.
